# Research status and hotspots of the systemic immune-inflammation index: A bibliometric analysis

**DOI:** 10.1097/MD.0000000000046220

**Published:** 2026-05-08

**Authors:** Miaomiao Yu, Ying Bai, Xinzhu Guo, Hao Wang

**Affiliations:** a Department of Intensive Care Unit, Beijing Jishuitan Hospital, Capital Medical University, Beijing, China.

**Keywords:** bibliometric, CiteSpace, prediction, prognosis, systemic immune-inflammation index, visualization analysis, VOSviewer

## Abstract

**Objective:**

The systemic immune-inflammation index (SII) has been linked to outcomes in various malignancies and has demonstrated utility in the diagnosis and prognosis of many conditions. This biomarker is gaining increasing recognition in the medical community. Understanding its developmental trajectory, critical areas of research, and future directions is essential for advancing its clinical application. However, there has been no literature bibliometric analysis on the SII. This study intended to visually analyze the research status and hotspots related to the SII.

**Methods:**

We utilized the Web of Science database to retrieve literature pertaining to the SII. Annual publication trends were statistically analyzed using Excel, VOSviewer, and CiteSpace for the visual analysis of authorship, institutional affiliations, countries, keywords, and co-cited references.

**Results:**

Two thousand seven hundred sixty-three publications were included. Fifteen thousand one hundred forty-eight authors contributed to these publications; 726 core authors represented 4.79% of the total. The leading countries were China (1532), Turkey (641), USA (91), Italy (86), and Japan (67), with the United States displaying the highest centrality score of 0.55. The top 3 institutions that contributed to the literature were the University of Health Sciences, Capital Medical University, and Sichuan University. Keyword analysis indicated that the research hotspots predominantly focused on tumors and their prognosis. Co-citation clustering revealed evolving themes, including prognostic significance, national health, myocardical infarction, covid-19 patient and disease activity, and multicenter study.

**Conclusion:**

The SII has garnered significant attention as a novel inflammation-related biomarker. However, further longitudinal and cross-sectional studies should be conducted to expand our understanding of its applications.

## 1. Introduction

The systemic immune-inflammation index (SII) is a novel inflammatory biomarker introduced by Professor Jia Fan and his team at Zhongshan Hospital, Fudan University, Shanghai, in 2014.^[1]^ The SII is based on peripheral blood parameters and calculated by the formula: SII = (platelet count × neutrophil count)/lymphocyte count. Initially identified as a prognostic indicator for hepatocellular carcinoma,^[1]^ the SII has since been linked to outcomes in various malignancies, including reproductive system tumors (germ cell tumor,^[2]^ prostate cancer,^[3]^ and cervical cancer^[4]^), digestive system tumors (esophageal squamous cell carcinoma,^[5,6]^ pancreatic cancer,^[7]^ and rectal cancer^[8]^), and lung cancer.^[9,10]^ Beyond oncology, the SII has demonstrated utility in the diagnosis and prognosis of both inflammatory and noninflammatory conditions, such as cerebral infarction,^[11]^ sepsis,^[12]^ cardiovascular diseases,^[13,14]^ and depression.^[15]^ This biomarker, characterized by its simplicity and accessibility, is gaining increasing recognition in the medical community.

The scientific knowledge graph, first proposed in 2003, not only visualizes knowledge but also serializes its lineage. This illustrates the development process, structure, and relationships within a specific knowledge domain. CiteSpace and VOSviewer are 2 commonly used software tools for knowledge graph visualization. VOSviewer employs a distance-based visualization method that is highly effective in presenting knowledge graphs. In contrast, CiteSpace uses clustering algorithms to detect research hotspots within disciplines, identify emerging terms, and assist researchers in identifying research trends and new directions. Understanding its developmental trajectory, critical areas of research, and future directions is essential for advancing its clinical application. However, there have been no literature bibliometric analysis on the SII. Thus, this study aimed to use VOSviewer and CiteSpace to visually analyze SII-related research and explore the development context, research status, hotspots, and trends in this field, thereby enhancing references for future research.

## 2. Methods

### 2.1. Data retrieval and download

**Figure 1.**
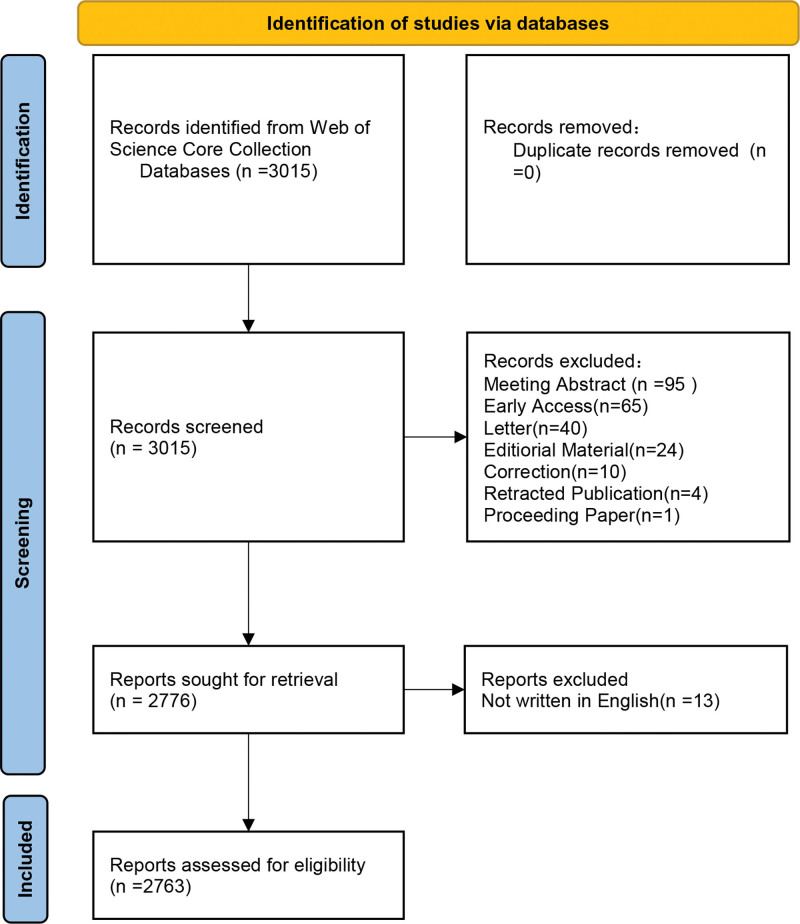
The flow chat of study screening.

We researched articles published between January 1, 2014 and August 1, 2025 in the Web of Science Core Collection. The retrieval formula used was TS = “systemic immune-inflammation index” OR “systemic immune-inflammatory index” OR “systemic immune inflammation index” with the document type set to “article” or “review artical” and the language to “English.” The flow chart of the literature screening process is presented in Figure 1.

### 2.2. Literature processing and data analysis

Excel 2013 was used to create a line chart of the number of documents published each year. The retrieved and processed data were then imported into VOSviewer (version 1.6.20, Centre for Science and Technology Studies, Leiden University, Leiden, The Netherlands) software for visual analysis of the publishing country, institution, author, and keywords and to create the corresponding visualization maps. The exported plain text file was renamed to “download_***” and imported into CiteSpace (version 6.4. R1, Drexel University, Philadelphia). The range was set from January 2014 to August 2025, with a time span of 1 year. The g-index method was selected using a *k*-value of 25. A keyword co-occurrence analysis was performed to identify keyword bursts. CiteSpace software was also used to analyze and cluster the co-cited documents and to create a clustering line graph.

## 3. Results

### 3.1. Analysis of annual publications

**Figure 2.**
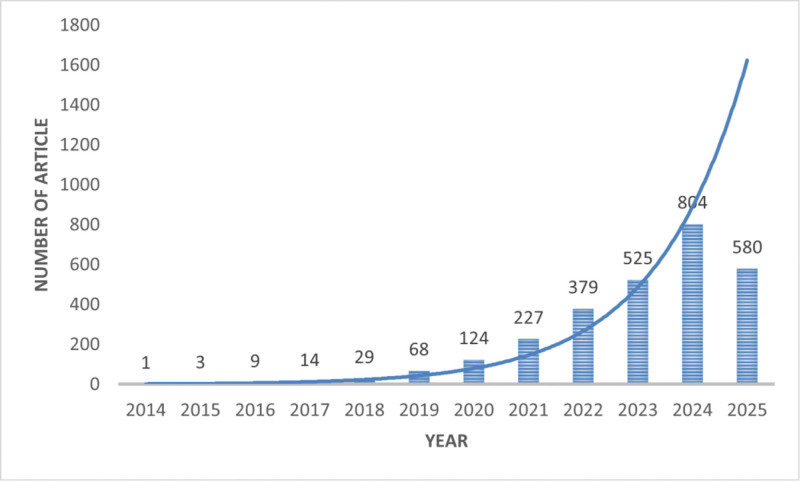
Annual publication trend chart.

A total of 2763 articles published between January 1, 2014 and August 1, 2025 were retrieved. Figure 2 shows the number of publications in each year. Since the introduction of the SII in 2014, the number of related publications has shown a consistent upward trend.It should be noted that the count for 2025 is 580, as the data collection was finalized on August 1, 2025. An analysis of the number of published articles from 2014 to 2024 reveals an exponential increase. This growth can be modeled by the function *Y* = 1.3 · (1.68) × (*R*-squared = 0.96).

### 3.2. Research power distribution analysis

#### 3.2.1. Author analysis

**Figure 3.**
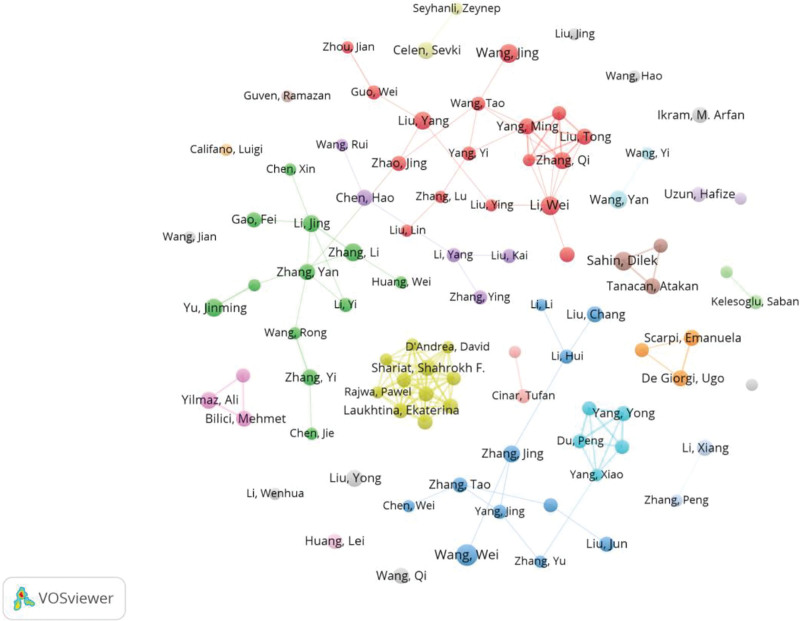
Author co-occurrence map. Each node represents an author. The size of the node indicates the number of publications, with larger nodes representing more publications. The node color indicates different clusters. The lines between nodes represent cooperative relationships between authors, with thicker lines indicating closer cooperation.

VOSviewer was used to analyze the authors of the 2763 included papers, revealing a total of 15,148 authors. The author with the highest number of publications had 15 papers. According to Price law, *m* = (Nmax) 1/2 × 0.749, and the minimum number of papers by core authors was calculated to be *m* = 2.901. Therefore, authors who published 3 or more papers were considered core authors in this field. There were 726 core authors, accounting for 4.79% of all authors, and they collectively published 761 papers, representing 27.54% of the total papers. Figure 3 shows the core authors’ information and collaborations. Table 1 listed the authors and their publication counts who had published >10 articles. Among authors with >10 published articles, 11 (78.57%) are from China and 3 (21.43%) are from Turkey. The author collaboration network revealed the formation of several clusters centered around highly productive authors. However, these collaborations were primarily small-scale and have not evolved into extensive international networks.

**Table 1:** Information of the top 14 authors of systemic immune-inflammation index-related publications.

Authors	Number of publications	Total citation	Country
Wang, Wei	15	154	China
Li, Wei	12	110	China
Sahin, Dilek	12	105	Turkey
Wang, Jing	12	137	China
Liu, Yang	11	296	China
Wang, Yan	11	138	China
Yu, Jinming	11	254	China
Zhang, Li	11	211	China
Celen, Sevki	10	49	Turkey
Li, Jing	10	192	China
Liu, Yong	10	91	China
Yilmaz, Ali	10	222	Turkey
Zhang Jing	10	123	China
Zhang Qi	10	249	China

#### 3.2.2. Analysis of countries and institutions

**Figure 4.**
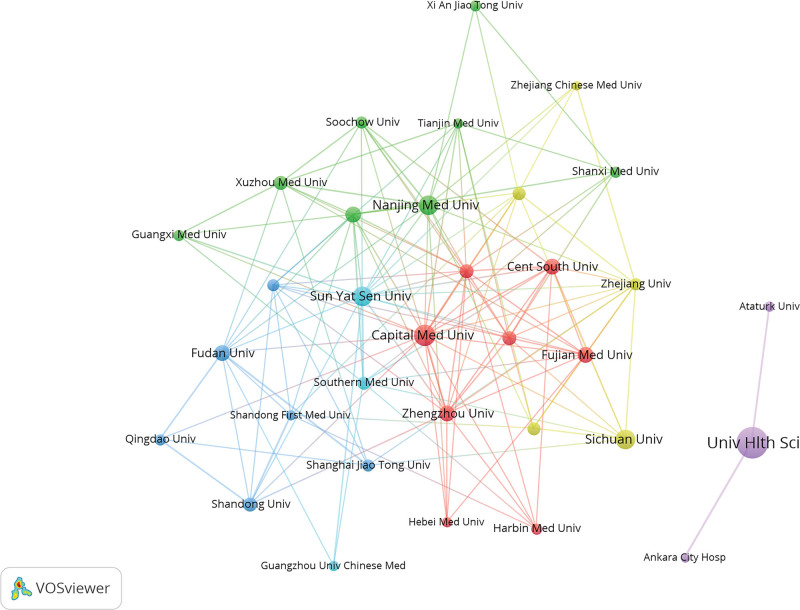
Country visualization map. Each node represents a country. The size of the node indicates the number of publications, with larger nodes representing more publications. The node color represents different clusters. Lines between nodes indicate cooperative relationships between countries, with thicker lines indicating closer cooperation.

**Figure 5.**
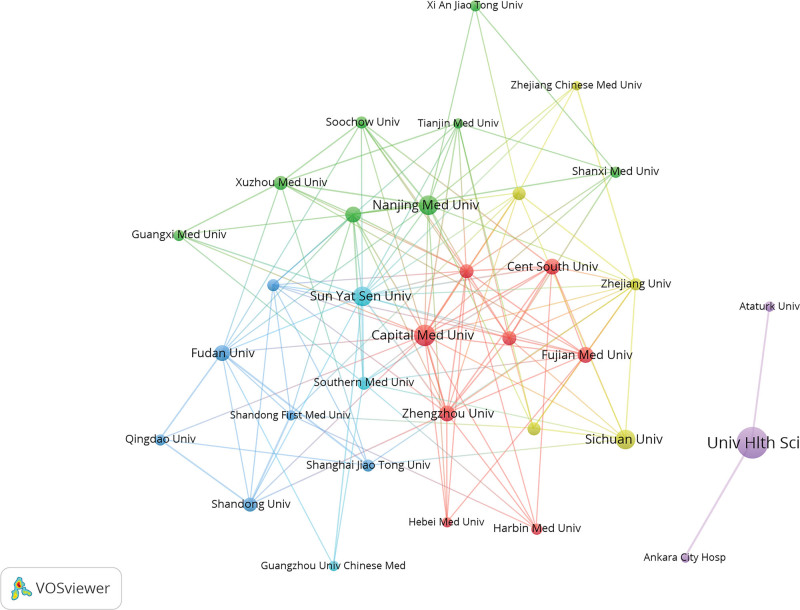
Research institutions visualization map. Each node represents an institution. The size of the node indicates the number of publications, with larger nodes representing more publications. The node color represents different clusters. Lines between nodes indicate cooperative relationships between institutions, with thicker lines indicating closer cooperation.

The VOSviewer analysis showed that researchers from 79 countries have published papers related to the SII. Authors from 38 countries published >5 papers. The top 5 countries were China (1532), Turkey (641), USA (91), Italy (86), and Japan (67). China had the most papers, accounting for 56.17% of all published documents (Table 2). Italy has the highest average citation counts (37.27). Some countries had formed cooperative relationships in SII research; however, others, such as Turkey, Pakistan, Slovekia, Slovenia still lacked international cooperation (Fig. 4). The top 3 institutions with the most SII-related publications were the University of Health Sciences, Capital Medical University, and Sichuan University. Figure 5 illustrated the collaborative relationships among institutions with >20 published articles. There are 33 institutions, forming 6 distinct collaboration clusters. These clusters are centered around the following institutions: University of Health Sciences (Turkey), Capital Medical University (China), Nanjing Medical University (China), Sichuan University (China), Fudan University (China), and Sun Yat-Sen University (China).The collaborative networks are primarily concentrated within China, demonstrating close cooperation among Chinese institutions. Similarly, the Health Sciences University has established collaborations with 2 other institutions in Turkey. It is noteworthy that these collaborative relationships are predominantly domestic, with a notable lack of international cooperation. Table 3 listed institutions that had published >40 papers.

**Table 2:** Top 5 countries by number of systemic immune-inflammation index-related publications.

Country	Number of publications	Total citation	Average citation	Centrality
China	1532	24,406	15.93	0.23
Turkey	641	4860	7.58	0.12
USA	91	1779	19.55	0.55
Italy	86	3205	37.27	0.09
Japan	67	996	14.86	0.03

**Table 3:** Research institutions with >40 systemic immune-inflammation index-related publications.

Institutions	Country	Number of publications	Centrality
University of Health Sciences	Turkey	150	0.33
Capital Medical University	China	78	0.11
Sichuan University	China	70	0.01
Nanjing Medical University	China	67	0.03
Sun Yat Sen University	China	66	0.05
Fudan University	China	49	0.01
Center South University	China	48	0.04
Zhengzhou University	China	48	0.04
Anhui Medical University	China	47	0.02
Fujian Med University	China	46	0.02
Xuzhou Med University	China	41	0.01

According to the calculation of intermediary centrality by CiteSpace, the centralities of the United States, China, and Turkey were all >0.1 regarding country publications. The United States had the highest centrality (0.55), followed by China at 0.23 and Turkey at 0.12. In terms of institutional publications, both the University of Health Sciences and Capital Medical University had centrality >0.1.

### 3.3. Knowledge base and research frontiers

#### 3.3.1. Keyword co-occurrence analysis

**Figure 6.**
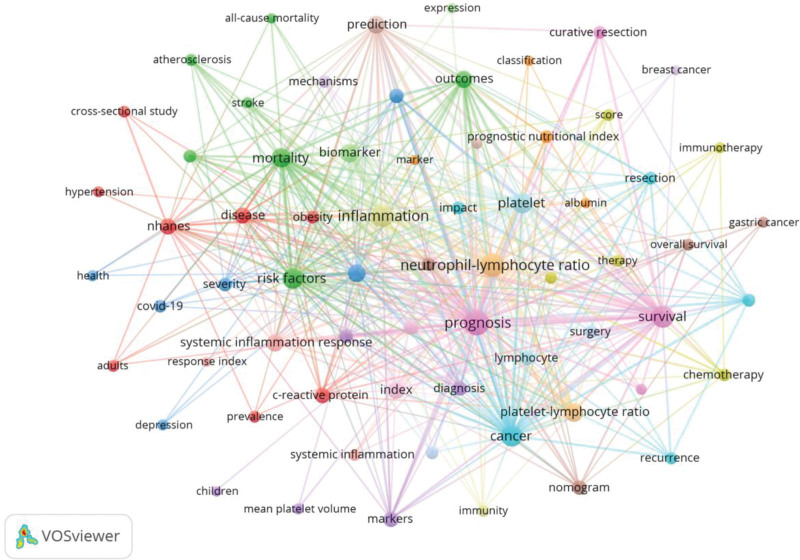
Keyword co-occurrence map. Each node represents a keyword. The size of the node indicates the frequency of occurrence, with larger nodes representing more frequent occurrences. The node color represents different clusters. The thickness of the lines connecting the nodes indicates the strength of the association between keywords.

Keyword co-occurrence analysis was conducted using VOSviewer. Synonymous keywords were combined, and the keyword “systemic immune inflammation index” was removed. The top 10 high-frequency keywords are listed in Table 4. A visualization map of keyword co-occurrence was shown in Figure 6.

**Table 4:** Top 10 high-frequency keywords in systemic immune-inflammation index-related publications.

Keyword	Frequency
Prognosis	761
Neutrophil–lymphocyte ratio	575
Inflammation	505
Survival	465
Cancer	409
Risk factors	370
Platelet	359
Mortality	321
Platelet–lymphocyte ratio	264
Biomarker	260

#### 3.3.2. Keyword burst analysis

**Figure 7.**
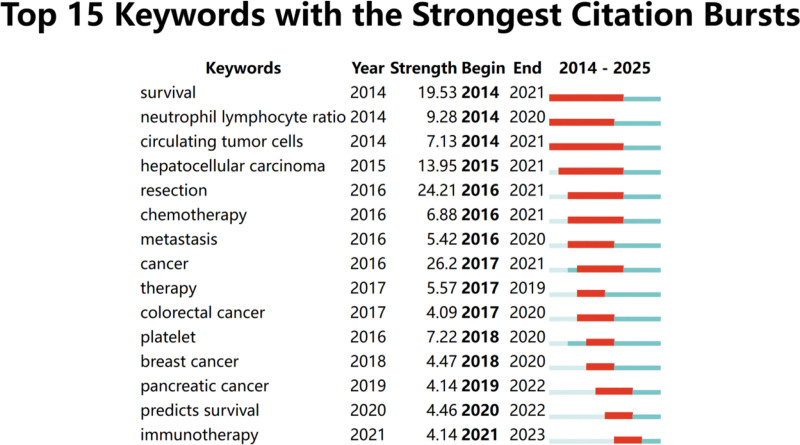
Keyword burst analysis.

CiteSpace was employed to analyze keyword bursts, identifying the top 15 keywords with the highest bursts (Fig. 7). Early burst keywords included survival, neutrophil lymphocyte ratio (NLR), circulating tumor cells, hepatocellular carcinoma, chemotherapy, metastasis, cancer, therapy, colorectal cancer, platelet, and breast cancer. Keywords that burst in the past 5 years included pancreatic cancer, predicts survival, and immunotherapy.

#### 3.3.3. Analysis of co-cited documents

**Figure 8.**
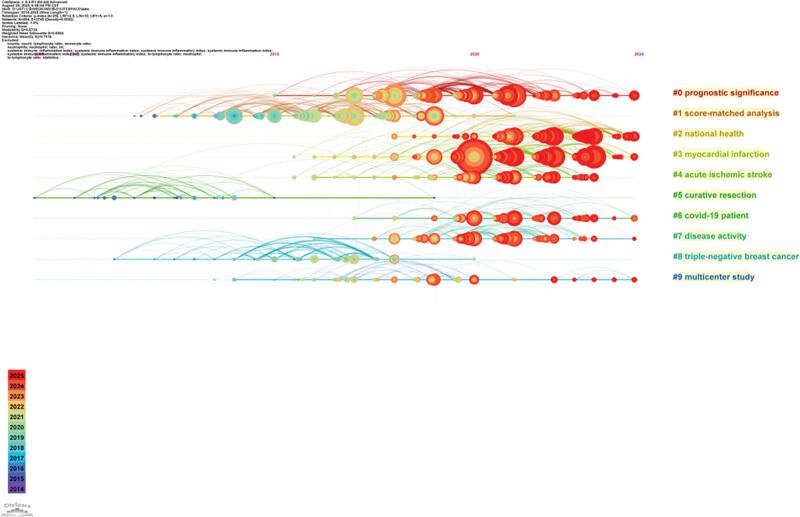
Time diagram of co-cited references.

A co-citation analysis of the 2763 retrieved literature items was conducted using CiteSpace. The top 10 most highly cited articles were presented in Table 5, and clustering was performed using the log-likelihood rate algorithm. A total of 13 clusters were generated, with the top 10 clusters retained and arranged by size from largest to smallest, as follows: prognostic significance, score-match analysis, national health, myocardial infarction, acute ischemic stroke, curative resection, covid-19 patient, disease activity, triple-negative breast cancer, and multicenter study (Table 6). Each cluster exhibited a silhouette coefficient of >0.7, indicating efficient clustering and reliable results. Among the top 10 most highly cited articles, 4 belonged to cluster 0 (prognostic significance), 4 belonged to cluster 3 (myocardial infarction). A theme words cluster analysis was carried out on co-cited literatures, and a time plot was drawn (Fig. 8). Cluster 0 (prognostic significance), cluster 2 (national health), cluster 3 (myocardical infarction), cluster 6 (covid-19 patient), cluster 7 (disease activity), and cluster 9 (multicenter study) were all continuously evolving, indicating that relevant studies were still ongoing.

**Table 5:** Top 10 highly cited systemic immune-inflammation index-related publications.

Publication	Citation frequency	Source	Intermediation centrality	Year	Cluster
Systemic immune-inflammation index (SII) predicted clinical outcome in patients with coronary artery disease	327	Eur J Clin Invest	0.00	2020	3
Global Cancer Statistics 2020: GLOBOCAN estimates of incidence and mortality worldwide for 36 cancers in 185 countries	155	CA-Cancer J Clin	0.04	2021	0
Prognostic value of systemic immune-inflammation index in cancer: a meta-analysis	151	J Cancer	0.01	2018	0
Prognostic value of preoperative systemic immune-inflammation index in patients with cervical cancer	143	Sci Rep-UK	0.02	2019	0
Systemic immune inflammation index (SII), system inflammation response index (SIRI) and risk of all-cause mortality and cardiovascular mortality: a 20-year follow-up cohort study of 42,875 US adults	140	J Clin Med	0.03	2023	3
Systemic immune-inflammation index for predicting prognosis of colorectal cancer	118	World J Gastroentero	0.01	2017	1
Systemic immune-inflammation index as a potential biomarker of cardiovascular diseases: A systematic review and meta-analysis	115	Front Cardiovasc Med	0.02	2022	3
The systemic-immune-inflammation index independently predicts survival and recurrence in resectable pancreatic cancer and its prognostic value depends on bilirubin levels: a retrospective multicenter cohort study	107	Ann Surg	0.13	2019	1
Systemic inflammation markers and cancer incidence in the UK Biobank	104	Eur J Epidemiol	0.01	2021	0
The associations of 2 novel inflammation indexes, SII and SIRI with the risks for cardiovascular diseases and all-cause mortality: a 10-year follow-up study in 85,154 individuals	102	J Inflamm Res	0.01	2021	3

**Table 6:** Top 10 clusters

Cluster number	Cluster size	Silhouette coefficient	Cluster name	Year
0	154	0.761	Prognostic significance	2020
1	112	0.836	Score-match analysis	2016
2	111	0.797	National health	2022
3	95	0.825	Myocardial infarction	2021
4	59	0.874	Acute ischemic stroke	2020
5	54	0.972	Curative resection	2012
6	50	0.906	Covid-19 patient	2020
7	48	0.897	Disease activity	2020
8	44	0.974	Triple-negative breast cancer	2015
9	39	0.81	Multicenter study	2018

## 4. Discussion

### 4.1. General information

Since the introduction of the SII in 2014, the number of SII-related published papers has gradually increased annually. The growth process can be divided into 2 stages. From 2014 to 2018, there was a slow initial phase characterized by limited attention and research on the SII, resulting in a small number of publications and a slow growth rate. Since 2019, however, there has been rapid expansion, with the number of papers nearly doubling, indicating increasing attention and deepening research in the field and signaling ongoing development.

China has led the number of publications, contributing to over half of the total papers. However, in terms of centrality, the United States exhibited the highest influence, followed by Turkey, highlighting their significant roles and collaborations in the field. Among the research institutions, the University of Health Sciences published the most papers and the highest centrality, indicating its pivotal role and extensive collaboration with other institutions. The analysis of the collaboration networks among countries, institutions, and authors revealed that current research on the SII was characterized by small-scale, localized cooperation. Collaboration was largely confined to domestic institutions and authors, with a notable lack of international partnerships. This pattern may be attributed to the nascent and exploratory nature of SII-related research. Since SII is easily and inexpensively obtained, any medical institution with a basic laboratory can readily conduct relevant studies. This low barrier to entry may, to some extent, reduce the necessity for complex, high-cost international collaborations.

A co-cited literature analysis can identify important literature in a field by analyzing the references of published literature, reflecting the knowledge base of the research field. According to our CiteSpace analysis, the article with the highest citation frequency was “Systemic immune-inflammation index (SII) predicted clinical outcome in patients with coronary artery disease” which was published in 2020 by Professor Yang Ya-Ling team at the Healthcare and Management Center in China, Taipei. Studies have shown that the SII has a good predictive effect on major cardiovascular events in patients with coronary artery disease after coronary intervention in patients with coronary artery disease.^[16]^ A co-citation analysis clustered the literature, and among the 10 most highly cited articles, 4 belonged to cluster 0 (prognostic significance), 4 to cluster 3 (myocardial infarction), and 2 to cluster 1 (score-match analysis). This distribution indicated that research related to the SII was primarily concentrated in the fields of oncology and cardiovascular diseases. The analysis also revealed that propensity score matching was a frequently employed statistical method within these studies.

Keywords reflect the core and main contents of an article. An analysis of high-frequency keywords reveals a focus on the main disease areas related to the SII, such as cancer. The keywords also highlight key applications, including prognosis, survival, risk factors, mortality, and biomarkers, as well as other related inflammatory markers like the NLR and platelet-lymphocyte ratio (PLR). Keyword burst analysis assesses dynamic changes and the trend of research direction by detecting the frequency change of a certain keyword within a certain period. Most of the mutation keywords stopped evolving before 2021. However, the burst keywords in the 5 most recent years included pancreatic cancer, predicts survival, and immunotherapy, which represent the frontier of SII-related research.

### 4.2. The clinical application of SII in major disease fields

#### 4.2.1. SII in oncology: a core marker for prognostic assessment and treatment guidance

In recent years, SII has been widely validated as an independent prognostic factor for various malignancies, demonstrating significant value in prognostic assessment and predicting treatment efficacy. Fan et al found that SII is an independent predictor of overall survival rate and relapse-free survival, and the increase of SII is correlated with vascular invasion, tumor volume increase, and early recurrence.^[1]^ A meta-analysis of 27 studies revealed that a high SII value was associated with a poor prognosis in patients with colon cancer, leading to significantly poorer outcomes in terms of overall survival, progression-free survival, disease-free survival, and recurrence-free survival.^[17]^ Similarly, a strong association has been observed between high pretreatment SII levels and a poor prognosis in various solid tumors, including gastric cancer, esophageal squamous cell carcinoma, urinary system tumors, prostate cancer, small-cell lung cancer, non-small cell lung cancer, and melanoma.^[3,18]^ This association has been consistently demonstrated across multiple cancer types.^[18]^ The prognostic value of the SII in tumors can be explained by the function of platelets, neutrophils, and lymphocytes. Platelets promote tumor angiogenesis and metastasis and protect tumor cells from antitumor immune responses. Neutrophils play an important role in tumor proliferation and metastasis by releasing inflammatory mediators, such as neutrophil elastase, interleukin-8, and matrix metalloproteinase-9. Moreover, lymphocytes prevent tumor growth and metastasis by promoting an antitumor immune response.^[19]^

In addition, SII level can reflect a patient’s response to treatment. In breast cancer patients undergoing neoadjuvant chemotherapy, baseline pretreatment SII levels were found to be closely related to treatment efficacy. Specifically, patients with lower baseline SII levels had a higher chance of achieving pathological complete response (16.4% vs 9.2%).^[20]^ Conversely, high SII levels are considered a risk factor for treatment failure and postoperative recurrence,^[6,8,21,22]^ suggesting that a more aggressive or intensive treatment regimen may be needed for these patients to improve their prognosis.

The SII also shows great potential in predicting patients’ response to immune checkpoint inhibitors.^[23]^ Among patients with advanced non-small cell lung cancer receiving anti-programmed cell death protein-1/programmed death-ligand 1 therapy, those who with a lower SII value achieved better treatment responses and survival outcomes.^[24]^ This predictive capability makes SII a potential tool for precision medicine. By stratifying patients based on their pretreatment SII levels, clinicians can better identify those who are likely to benefit from immunotherapy or design a more aggressive combination therapy for high-risk patients with a high SII, thereby achieving a more precise treatment plan.

#### 4.2.2. Cardiovascular diseases: risk prediction and stratification

In the risk assessment of acute pulmonary embolism, the SII has been proven to have significant value in the diagnosis and risk stratification of high-risk pulmonary embolism.^[25,26]^ A retrospective study found that SII in the pulmonary embolism group was significantly higher than that in control group, and the SII was identified as one of the effective markers for predicting high-risk pulmonary embolism during risk stratification.^[8]^ Other studies have also shown a significant correlation between a high SII and in-hospital mortality in pulmonary embolism, suggesting its potential as a predictor of in-hospital mortality.^[27]^

The prognostic value of the SII has also been validated in the field of coronary artery disease. A study showed that in ICU patients with acute myocardial infarction and hypertension, a high SII was identified as an independent risk factor for all-cause mortality at 30 and 365 days and were associated with an increased risk of severe complications such as heart failure and cardiogenic shock.^[28]^ An elevated SII is also associated with major adverse cardiovascular events following coronary intervention.^[16]^ In a long-term follow-up of patients with three-vessel coronary artery disease who underwent revascularization, a high SII was found to be an independent risk factor for major adverse and cerebrovascular events and death. Combining the SII with traditional risk factors can enhance predictive capabilities.^[14]^ Furthermore, a high SII is also associated with poor prognosis in heart failure and valvular heart disease.^[29,30]^

The SII has also been used for comprehensive risk stratification in cardiovascular diseases. Research had shown a significant negative correlation between SII levels and the American Heart Association’s “Life’s Essential 8” health score,^[31]^ suggesting that a healthy lifestyle (e.g., controlling weight and blood sugar, not smoking) may lower the SII, thereby ensuring cardiovascular health. The SII was positively correlated with the traditional Framingham cardiovascular risk score, indicating that it can serve as an effective index for assessing a person’s 10-year CVD risk and providing a reference for risk stratification and the formulation of preventive strategies.^[32]^

#### 4.2.3. The application of SII in other diseases

The SII can reflect the inflammatory state of the body, the neutrophil count reflects the acute and chronic inflammatory responses and a weakened immune response when their count is reduced. In addition, platelets play a crucial role in the immune system and inflammation as they release cytokines and chemokines that contribute to the development of chronic inflammation. Platelets can also interact with white blood cells, such as neutrophils and monocytes, to enhance their inflammatory response.^[33,34]^ Therefore, the SII can reflect the balance between inflammation and immunity throughout the body.

SII also demonstrates significant diagnostic and prognostic value in a variety of diseases, including autoimmune disorders, neurological diseases, infectious diseases, and so on.

In the fields of autoimmune and rheumatic diseases, elevated SII level showed a significant positive correlation with disease activity in patients with systemic lupus erythematosus. The index can be used to track overall disease activity and may also predict specific adverse events such as lupus nephritis and unfavorable pregnancy outcomes.^[35,36]^ A correlation between SII and disease activity had also been established in conditions such as rheumatoid arthritis, spondyloarthritis, psoriasis, and psoriatic arthritis.^[36]^

A high SII at admission is a significant and independent predictor of increased short-term mortality in sepsis patients, with a pooled risk ratio of 1.51.^[37]^ Similarly, in COVID-19 patients, elevated SII levels upon admission are closely associated with disease severity and mortality.^[38]^ Studies have also found that the SII is associated with all-cause mortality. Xia et al found that in older individuals (>60 years), the SII showed a strong association with all-cause mortality.^[39]^

### 4.3. Comparative analysis of SII and other biomarkers

In addition to SII, many other peripheral blood-based inflammatory indicators have been widely adopted in clinical practice. The most common among these are the NLR, PLR, monocyte-to-lymphocyte ratio (MLR), and systemic inflammatory response index (SIRI). The NLR is calculated from the ratio of absolute peripheral blood neutrophil and lymphocyte counts. The PLR is calculated from the ratio of peripheral blood platelet count to lymphocyte count. The MLR is calculated as the ratio of peripheral blood monocyte to lymphocyte counts, while the SIRI is obtained by calculating (neutrophil count × monocyte count)/lymphocyte count from peripheral blood.^[40]^ Although these inflammatory indicators are all derived from a complete blood count, they reflect the balance of the inflammatory and immune systems from different perspectives due to the different factors they incorporate.

In studies predicting the prognosis of patients with bloodstream infections, SII and NLR showed superior predictive efficacy for mortality risk compared to PLR, SIRI, and MLR.^[40]^ In contrast, SIRI, NLR, and PLR demonstrated greater value in predicting the short-term prognosis of acute ischemic stroke.^[41]^ A study investigating inflammatory markers and prognosis in ST-segment elevation myocardial infarction found that high NLR, SII, and SIRI were all independent risk factors for 1-year mortality, but only NLR was associated with reinfarction in a dose-dependent manner.^[42]^ These findings demonstrate that different inflammatory indicators offer unique advantages depending on the specific disease. Future research could focus on identifying new, more comprehensive composite indicators or effectively combining existing ones to enhance their clinical utility.

### 4.4. Combined application of SII and prognostic nutritional index (PNI)

The prognostic nutritional index (PNI) is a commonly used indicator based on serum albumin and lymphocyte count to assess a patient’s nutritional status and immune function. It is widely applied to evaluate perioperative complications and prognosis in patients with malignant tumors.^[43]^ The combined use of SII and PNI integrates comprehensive indicators of nutrition, immunity, and inflammation, and has demonstrated significant value in prognostic assessment in clinical practice. Among patients with solid tumors, those with a high SII combined with a low PNI showed the worst clinical outcomes.^[43]^ A study on the prognosis of patients with advanced non-small cell lung cancer receiving platinum-based doublet chemotherapy found that the SII–PNI score was closely related to patient overall survival.^[44]^ SII and PNI are also associated with the prognosis of early gastric cancer, and a combined score of PNI, SII, and lymph node metastasis was able to predict performance.^[45]^ The combined PNI–SII score at admission was closely related to the prognosis of patients with severe community-acquired pneumonia, with a high score being an independent factor for predicting a high 28-day mortality risk.^[46]^ These findings suggest that the combined SII–PNI score can effectively identify high-risk populations and reflects a general systemic pathological state related to disease severity.

### 4.5. Challenges and breakthrough directions of SII

Although SII has been widely investigated in clinical settings, several issues are still unresolved. Currently, most studies are retrospective, single-center, and small sample sizes.^[47]^ An analysis of author, country, and institutional collaborations reveals a lack of large-scale, multicenter, and international studies. The absence of a standardized cutoff value for SII makes it challenging for clinicians to directly apply research findings to clinical diagnosis.^[45,47,48]^

While SII exhibits high sensitivity, its specificity is low. Elevated SII levels are observed in various pathological conditions, which hinders accurate disease determination based solely on an increase in SII. Consequently, SII is currently used only as an auxiliary reference.

Given these limitations, future research on SII should focus on the following aspects:

Firstly, large-scale, multicenter, prospective studies are needed, which can mitigate selection bias and explore the universal applicability of SII across different populations and disease subtypes. Secondly, standardization and cutoff value exploration are requested, establishing a unified SII cutoff value or developing a system of cutoff values for different diseases and populations. Thirdly, a deeper exploration of the specific biological mechanisms linking elevated SII to disease progression and poor prognosis remains a critical future direction. Lastly, future efforts may involve developing composite scoring systems that incorporate a broader range of peripheral blood parameters, serum biomarkers, and even molecular markers to achieve more precise individualized prediction. The combined application of such biomarkers may improve the accuracy of disease diagnosis and prognosis prediction, which is also one of the future research directions.

This study had limitations. First, the core data set of WOS is used as the data source in this study. Second, subsequent articles in other databases and languages should have been included for further analysis. This may result in an incomplete list of included studies and some bias in the findings.

In conclusion, the SII, a novel inflammation-related biomarker, has attracted wide attention from researchers owing to its convenience, accessibility, and wide applicability. Most studies have focused on tumor prognosis, metastasis, and therapeutic effects. In recent years, the SII has also been applied to various diseases, but it is still necessary to expand the research scope vertically and horizontally and deepen the clinical application and understanding of the SII, prediction model and its combined application with other biomarkers may be the direction of future research. By using VOSviewer and CiteSpace bibliometrics software, this article makes a visual analysis of SII-related literature, which helps researchers quickly understand the distribution of major research focuses, research hotspots, and future development trends in this field and provides certain references for subsequent research.

## Acknowledgments

We appreciate the free use of CiteSpace and VOSviewer software.

## Author contributions

**Conceptualization:** Miaomiao Yu, Hao Wang.

**Formal analysis:** Ying Bai.

**Investigation:** Miaomiao Yu, Ying Bai.

**Methodology:** Ying Bai, Xinzhu Guo.

**Software:** Miaomiao Yu, Xinzhu Guo.

**Writing – original draft:** Miaomiao Yu.

**Writing – review & editing:** Hao Wang.
